# Television viewing through ages 2-5 years and bullying involvement in early elementary school

**DOI:** 10.1186/1471-2458-14-157

**Published:** 2014-02-12

**Authors:** Marina Verlinden, Henning Tiemeier, René Veenstra, Cathelijne L Mieloo, Wilma Jansen, Vincent WV Jaddoe, Hein Raat, Albert Hofman, Frank C Verhulst, Pauline W Jansen

**Affiliations:** 1 The Generation R Study Group, Erasmus MC-University Medical Center, Rotterdam, The Netherlands; 2 Department of Child and Adolescent Psychiatry/Psychology, Erasmus MC-Sophia, University Medical Center Rotterdam, P.O. Box 2060, Rotterdam 3000 CB, The Netherlands; 3 Department of Epidemiology, Erasmus MC-University Medical Center, Rotterdam, The Netherlands; 4 Department of Psychiatry, Erasmus MC-University Medical Center, Rotterdam, The Netherlands; 5 Department of Sociology, University of Groningen, Groningen, The Netherlands; 6 Municipality of Rotterdam, Research and Business Intelligence, Rotterdam, The Netherlands; 7 Department of Public Health, Erasmus MC-University Medical Center, Rotterdam, The Netherlands; 8 Municipality of Rotterdam, Department of Social Development, Rotterdam, The Netherlands; 9 Department of Pediatrics, Erasmus MC-University Medical Center, Rotterdam, The Netherlands

**Keywords:** Television, Bullying, Aggression, Children

## Abstract

**Background:**

High television exposure time at young age has been described as a potential risk factor for developing behavioral problems. However, less is known about the effects of preschool television on subsequent bullying involvement. We examined the association between television viewing time through ages 2-5 and bullying involvement in the first grades of elementary school. We hypothesized that high television exposure increases the risk of bullying involvement.

**Method:**

TV viewing time was assessed repeatedly in early childhood using parental report. To combine these repeated assessments we used latent class analysis. Four exposure classes were identified and labeled “low”, “mid-low”, “mid-high” and “high”. Bullying involvement was assessed by teacher questionnaire (n = 3423, mean age 6.8 years). Additionally, peer/self-report of bullying involvement was obtained using a peer nomination procedure (n = 1176, mean age 7.6 years). We examined child risk of being a bully, victim or a bully-victim (compared to being uninvolved in bullying).

**Results:**

High television exposure class was associated with elevated risks of bullying and victimization. Also, in both teacher- and child-reported data, children in the high television exposure class were more likely to be a bully-victim (OR = 2.11, 95% CI: 1.42-3.13 and OR = 3.68, 95% CI: 1.75-7.74 respectively). However, all univariate effect estimates attenuated and were no longer statistically significant once adjusted for maternal and child covariates.

**Conclusions:**

The association between television viewing time through ages 2-5 and bullying involvement in early elementary school is confounded by maternal and child socio-demographic characteristics.

## Background

Bullying is conventionally defined as intentional and continuous peer aggression, involving power imbalance between a victim and aggressor [1]. It is a common problem in early elementary school. About 20-30% of children are involved in bullying either as a bully, victim or a bully-victim (i.e. being involved in bullying as a bully and a victim) [2,3]. Bullying involvement is associated with diverse behavioral and emotional problems in children [4]. Thus, identifying potential risk factors that may predispose children to bullying involvement at young age is important for informing prevention strategies.

Several bullying involvement roles are typically defined, among which the roles of a victim, bully and a bully-victim are of primary interest as these children are directly involved in bullying and are most at risk of psychopathology. For instance, victims often have internalizing problems and show increased symptoms of anxiety, depression, low self-esteem and poor social skills [4]. The behavior of bullies is marked by externalizing problems and it resembles behavior of children with conduct problems [4]. Furthermore, bullies typically demonstrate high levels of proactive aggression [5]. Bully-victims usually show high levels of both proactive and reactive aggression [6], and have symptoms of both internalizing and externalizing problems [7]. Compared to bullies and victims, the bully-victims stand-out as a group of children with the highest risk of developing multiple psychopathologic behaviors [8], and they are most likely to remain involved in bullying for prolonged periods of time [9]. It should be noted, the association between internalizing/externalizing problems and bullying involvement is most likely reciprocal. Studies showed that internalizing problems contribute to victimization, while being victimized in the first grades of elementary school uniquely contributes to an increase in internalizing and externalizing problems [7,10]. Furthermore, bullying involvement also increases the risk of later psychiatric disorders: in a large cohort study it was shown that being victimized at age 8 year predicts psychiatric disorders, such as anxiety and antisocial personality, 10 to 15 years later [11].

Exposure to media violence is considered to be one of the factors associated with aggressive and violent behavior [12,13]. Since Bandura’s classical studies [14] on child imitation of violent videos, various observational and experimental studies have provided an abundance of evidence for a relation between viewing violence in the media and high levels of aggressive behavior [13]. Besides linking young children’s viewing of violence on TV to aggression [15,16], studies also show a relation between adolescents’ violent video game play and aggressive behavior [17]. These findings can be explained by content-based theories that emphasize the importance of the content and quality of programs watched. Following the content-based approach, children learn from the observed content by using cognitive and social learning mechanisms, as was suggested by Bandura in the social learning theory of aggression [18]. Exposure to violent content on TV may influence children’s cognitive scripts and information processing, which then may impact children’s social problem solving and behavior. Children who are exposed to interpersonal or media violence are likely to encode and store cognitive rules on how to behave in problematic social situations, and these cognitions may guide their behavior in conflict situations [12]. Furthermore, children who are frequently exposed to violent television programs may become desensitized to aggression what, in its turn, can lead to weaker negative affective responses to observing violence and to stronger acceptance of aggressive behavior [12,13,19].

Some studies demonstrated the negative effects of the *time* of TV exposure on behavior [20-22]. This is in line with the time displacement theory that suggests that young children who are exposed to TV or screen media for excessively long periods of time are spending less time on intellectually and physically stimulating activities, as well as on peer interactions that are essential for the development of social skills. If parents of young children do not facilitate children’s engagement in extracurricular activities that stimulate children’s cognitive, physical and social development, children are likely to develop a passive lifestyle with television viewing as a default strategy of spending their time [23]. Following this view, a possible consequence of *excessive* TV exposure time at young age could include poor social skills and problems with peers.

Relatively little is known about the effects of TV viewing time on bullying involvement, particularly in young children. Because television exposure has been related to aggression, one may speculate that high television exposure at preschool age may predispose children to involvement in school bullying. However, another plausible assumption could be that children who are involved in bullying are likely to watch more television due to deprived relations with their peers. Studying television exposure at preschool age, prior to bullying occurrence, can reveal important information about children’s possible susceptibility to bullying involvement. Results of two earlier studies in young elementary school children suggested that duration of television exposure at young age can be a risk factor for bullying [24] and victimization [25]. In a longitudinal study of 1314 children in Canada [25], Pagani and colleagues found that child TV exposure at age 2.4 and 4.4 years predicted victimization by classmates at age 10 years. Also, TV exposure at age 4 years was associated with an elevated risk of bullying at age 6-11 years in a prospective study of 1266 children in the US [24]. However, the association between preschool television viewing and bullying involvement in early elementary school needs to be ascertained in other large population-based studies, using multiple assessments of exposure throughout early childhood and carefully examining the issue of potential confounding variables.

Furthermore, previous studies that examined the association between time of television viewing and bullying involvement in early elementary school, although they were well-conducted, had some limitations, e.g. they used either only teacher or maternal report to assess bullying [24,25]. Teachers and parents are not always aware of child bullying involvement, and in order to avoid this potential bias, information about child bullying involvement should be ideally based on reports of multiple informants. One of the measures of bullying involvement used in our study is based on a peer nomination method, and is a combination of child self-report and ratings by multiple peers. Obtaining information on bullying involvement from teachers and from multiple peers strongly enhances its reliability.

Importantly, previous studies in young children did not examine the effects of television exposure on specific bullying involvement roles (i.e. victim, bully, bully-victim) [24,25], while these roles may be associated with different risk factors and outcomes [26,27]. Also, in the existing studies, television exposure was assessed only at one [24] or two [25] time points, while multiple measurements of child TV exposure at preschool age provide more comprehensive information. Unlike a single assessment, which generates information about the exposure at one particular point in time, repeated assessments capture the patterns of the exposure over time. Finally, the role of other underlying factors should be considered as a possible alternative explanatory mechanism. Several socio-demographic and psychosocial covariates that may confound the association between television viewing and consequent bullying problems were selected in our study based on previous studies of television exposure in young children [22,24,25]. Analyses were adjusted for: child age, gender, national origin, internalizing and externalizing problems, and daycare attendance; maternal age, parity, educational level, marital status, household income, symptoms of depression, and parenting stress. We considered these potential confounders as conceptually relevant and examined whether inclusion of these variables in a model resulted in a change of the effect estimate of television viewing on bullying involvement. Importantly, apart from child and maternal socio-demographic characteristics, we considered child behavioral and emotional problems as possible confounding factors of the association between television viewing and bullying, as studies show that early television exposure is associated with behavioral problems [28,29], and that children’s internalizing and externalizing problems are associated with bullying involvement [7].

The objective of our study was to examine the association between television viewing time at ages 2-5 years and bullying involvement in grades 1-2 of elementary school. We aimed to extend research knowledge in this field by: using repeated assessments of TV exposure time at preschool age, examining different bullying involvement roles, and by accounting for possible confounding effects of child and maternal factors. Based on the findings from previous studies [24,25], we hypothesized that time of television exposure is associated with a higher risk of bullying and peer victimization. In addition to our main aim of studying the prospective association between the time of TV exposure and bullying involvement, we examined whether an exposure to violent content at age 5 years is associated with bullying involvement in early elementary school.

## Methods

### Design and study participants

Our study was embedded in the Generation R Study, a large population-based cohort of children in Rotterdam, the Netherlands. An extensive description of the cohort and various assessments that were carried out among children and their parents can be found elsewhere [30,31]. All participants provided written informed consent and the study has been approved by the Medical Ethics Committee of the Erasmus University Medical Centre.

**Figure 1:**
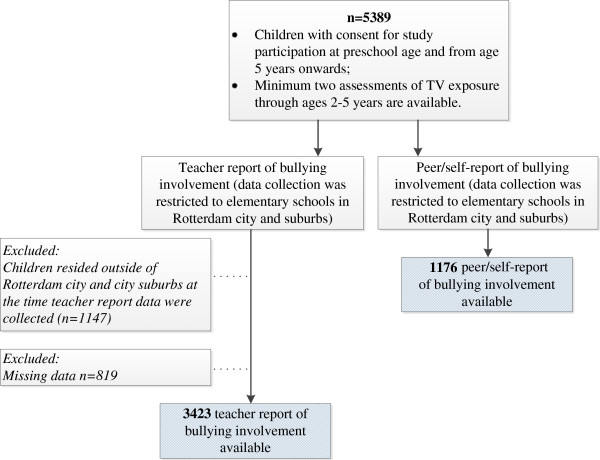
Flowchart of the sampling procedure.

Data on television exposure (i.e. minimum two assessments) throughout ages 2-5 were available for 5389 Generation R children. At the time Generation R participants attended grades 1-2 of elementary school, teachers were asked to fill out a questionnaire that included questions about child bullying involvement at school. The data collection was restricted to Rotterdam city and suburbs, thus teachers filled out questionnaires only for children residing in Rotterdam and suburbs (see Figure 1 for the flowchart of the sampling procedure). Teacher report of bullying was available for 3423 out of 5389 children with data on television viewing. Additionally, an extensive assessment of peer relationships at school, involving child peer- and self-reports, was performed in a subsample of the Generation R Study participants and their classmates. Peer/self-reports of bullying involvement were available for 1176 children. The two data collection procedures, i.e. teacher and peer/self-reports of bullying involvement, were collected as part of different assessments independently from one another. Consequently, the association between TV exposure through ages 2-5 and bullying involvement in early elementary school was studied in 3423 children using teacher report, and in 1176 children using peer/self-report of bullying involvement.

### Measures

#### TV exposure

At the ages 2, 3, 4 and 5 years children’s TV exposure time was assessed by parental questionnaires. At the youngest age, duration of *daily television viewing* was measured using the following answer categories: *“never”, “<0.5 hour”, “0.5-1 hour”* and *“>1 hour”*. Categories of TV exposure time at the ages 3, 4 and 5 years were modified (maximum exposure category *“>1 hour”* was adapted to: *“1-2 hours”* and *“>2 hours” *of daily viewing) to better differentiate at the higher ranges of TV viewing in older children. The four TV exposure measures were combined into a latent variable that reflects child TV viewing patterns throughout ages 2-5 years (see statistics section for the description of the method).

Our main analyses are focused on examining the effects of the time of TV exposure. In addition, following the above reviewed work of Bandura and others, we also examined the effect of exposure to violent television content on children’s bullying involvement. At the age of 5 years, parents of the children reported on whether their children were exposed to violent content on TV/video (*“yes/no”*).

#### Bullying involvement

Teachers rated children’s involvement in bullying (n = 3423, mean age 6.8 years) over past three months with regard to four types of bullying (physical, verbal, relational and material). To assess physical victimization teachers were asked: *“Was a child victimized physically by other children, for instance by being hit, kicked, pinched, or bitten?”*. Verbal victimization was measured by: *“Was a child victimized verbally, for instance by being teased, laughed at, or called names?”*. Relational victimization was assessed by: “*Was a child excluded by other children?*”. Lastly, material victimization was studied by the question: “*Were the belongings of a child hidden or broken by other children?*”. Bullying was measured using the same type of questions but then inquiring about a child’s behavior as a bully. For example, to assess physical bullying teachers were asked: *“Did a child physically bully other children, for instance by hitting, kicking, pinching, or biting them?”*. Items were rated on a four-point Likert scale with answer categories ranging from *“Never or less than once per month”* to *“More than twice per week”*. Based on these ratings we categorized children into four mutually exclusive groups: *“uninvolved in bullying”, “bullies”, “victims” and “bully-victims”*[2]. Children, whose behavior with regards to all bullying and victimization items was rated with *“Never or less than once per month”*, were categorized as *“uninvolved in bullying”*. Children were categorized as *“victims”* if teachers reported them being victimized at least once a month in any of the four forms of victimization. Similarly, children were categorized as “*bullies”* when a teacher reported their involvement as a bully in any form of bullying at least once a month. Children rated by teachers as both bullies and victims were categorized as “*bully-victims”*.

Children completed a computerized assessment, the PEERS Measure (n = 1176, mean age 7.6 years), during which they independently reported about their experience of peer victimization. Detailed description of the method can be found elsewhere [32]. Again, four questions were used to assess different forms of victimization: physical, verbal, relational and material. We used the peer nomination method: children nominated their classmates by clicking on their photos on the screen, in order to indicate by whom they were victimized. The number of nominations a child gave to others was used to calculate individual victimization scores. The nominations a child received from classmates were used to calculate individual bullying scores. Considering that on average a school class consisted of 21 children, each child’s bullying score was based on the rating of about 20 peers. Therefore, the bullying score of each child reflects the extent to which a child is perceived as a bully by his/her classmates. Higher scores represent more bullying/victimization nominations. The individual bullying and victimization scores across different forms of bullying and victimization were averaged to obtain the overall bullying and victimization scores. In order to define specific roles of children’s involvement in bullying, we dichotomized the continuous bullying and victimization scores using the top 25^th^ percentile as cut-off in the sample of all children who were assessed using the PEERS Measure. This cut-off was applied also in earlier studies that used the peer nomination method [33]. The dichotomized measures were then used to categorize children into the non-overlapping groups: *“uninvolved in bullying”, “bullies”, “victims”* and *“bully-victims”*.

### Covariates

Inclusion of the covariates resulted in a 5-10% change of the effect (inclusion of some resulted in a substantially larger change than 10%, e.g. maternal educational level, child ethnicity or household income). Although inclusion of few variables (namely, child age, gender, maternal depression symptoms, and parenting stress) led to a relatively small change of the effect estimates, all the variables were treated as potential confounders based on their conceptual relevance, and also, because in our data these covariates were associated with both children’s television exposure and with bullying involvement.

Information about child’s date of birth and gender was obtained from hospital registries. All other covariates were assessed using parental questionnaires. National origin of a child was defined by country of birth of the parent(s) and categorized as *Dutch*, *Other Western* or *Non-western.* Daycare attendance, assessed at age three years, was categorized as *“not attending daycare”* and *“attending daycare”*.

We also adjusted the analyses for child (pre-existing) internalizing and externalizing problems. Studies showed that these behavioral problems are associated with both television viewing and bullying involvement: children with behavioral problems are likely to watch more television [34] and children involved in bullying often show internalizing and externalizing problems [4,7,33]. The Dutch version of the Child Behavior Checklist (CBCL1½-5) [35] was used to obtain parent reports of children’s externalizing and internalizing behavioral problems at age 18 months. The 29-item externalizing scale of the CBCL consists of two subscales: Attention Problems and Aggressive Problems. The internalizing scale (36 items) consists of four syndrome scales: Emotionally Reactive, Anxious Depressed, Withdrawn and Somatic Complaints. The CBCL1½-5 has good reliability and validity [35].

Birth order (i.e. parity) was used to categorize children as “*first-born”* and “*not first-born*”. The highest attained *educational level* of the mother (4 categories) ranged from “*low”* (<3 years of general secondary education) to “*high”* (higher academic education/PhD) [36]. Marital status was dichotomized into: *“married/living together”* and *“single”*. The net monthly household *income* was categorized: *“below social security level”* (<1200 Euros), *“average”* (1200-2000 Euros) and *“modal”* (>2000 Euros). We used the Brief Symptom Inventory, a validated instrument containing 53 self-appraisal statements [37] to assess maternal symptoms of depression when children were 3 years old. Parenting stress was assessed when children were 18 months old, using the Nijmeegse Ouderlijke Stress Index–Kort [38], a questionnaire consisting of 25 items on parenting stress related to parent and child factors. For both measures, sum scores were used in the analyses.

### Statistical analyses

In order to combine the information about children’s TV exposures throughout ages 2, 3, 4 and 5 years, we used latent class analyses. A variable summarizing the pattern of TV exposure throughout preschool age carries more information than a single assessment at either of the different time points analyzed separately. Therefore, in the analyses we used a latent variable that combined information about child TV exposures at ages 2, 3, 4 and 5 years. However, we also studied the association using the separate TV exposure measurements at different ages to examine whether there is a specific vulnerable age at which viewing TV predisposes children to later risk of bullying involvement, and to ensure the reliability of our findings irrespective of the method.

TV exposure patterns throughout ages 2-5 years were identified using latent class analyses performed in Mplus (version 6.12). With this technique, latent classes (i.e. groups) of children were generated based on their TV exposures at four different ages. The number of latent classes was determined by assessing the model fit indices: Bayesian Information Criteria (BIC) and the Lo-Mendel-Rubin Likelihood Ratio-Test (LMR-LRT; see Additional file 1: Table S1), along with other relevant characteristics such as the size of groups. The latent classes were derived from the data of all Generation R participants with at least two TV exposure assessments available throughout ages 2-5 years (N = 5389). The identified classes were then analyzed as predictors of bullying involvement at school.

Teacher- and peer/self-reported data on bullying were analyzed separately using multinomial regression models. We examined whether latent classes of TV watching throughout ages 2-5 predicted bullying involvement in early elementary school either as a bully, victim or a bully-victim (reference group: uninvolved). Two models were examined: (1) unadjusted and (2) adjusted for socio-demographic and psychosocial covariates. We also adjusted the analyses for separate groups of covariates in different models to examine if any observed association was confounded by a specific combination of child or maternal factors. Examination of the correlation coefficients for particularly strong correlations between the individual covariates (i.e. above .80) that could lead to collinearity problems during estimation of regression coefficients, showed no indication for concern. Additional collinearity diagnostic analyses – calculation of the variance inflation factor (VIF) values for the control variables – did not raise any further concerns (mean VIF = 1.42, VIF values for individual covariates ranged from 1.01 to 1.93; against the value of VIF > 10 indicating possible collinearity problems)*.*

Missing data in the covariates were estimated using multiple imputation technique (chained equations). All covariates were used to estimate the missing values. The reported effect estimates are the pooled results of 30 imputed datasets. The imputed datasets were generated using STATA (Stata/SE 12.0, StataCorp LP Texas). In order to account for the clustered structure of the data (i.e. children from the same school classes were tested), we performed multinomial regression analyses using clustered robust standard errors (Huber-White method of variance estimation). School class was used as cluster variable.

### Characteristics of the retained sample

Of all children with information on TV exposure, we compared those with (n = 3423) and without (n = 1966) teacher-reported data on bullying involvement. Data were missing more often for children of Dutch and other Western national origin than for children of non-Western origin. Children without a teacher report on bullying had somewhat higher levels of parent-reported externalizing problems (mean score 7.44, SD = 6.61 vs. 6.91, SD = 6.08, p = 0.004) and were more likely to be categorized as belonging to the low or mid-low TV exposure class. Mothers of children with missing data on bullying involvement were more often higher educated (37.0% vs. 47.7%, p < 0.001), and had a higher household income (9.5% vs. 12.0%, p = 0.009) compared to those for whom data on TV exposure was available.

## Results

### Sample characteristics

Child and maternal characteristics of the study sample are presented in Table 1. Our sample comprised 50.6% boys and 63.1% children of Dutch national origin (Table 1). Based on teachers’ ratings, 69.1% of children were categorized as uninvolved in bullying, 14.7% as bullies, 4.1% as victims and 12.1% as bully-victims. Proportions of bullying involvement were slightly different in peer/self-reported data, with fewer children categorized as uninvolved (60.0%, p-value for comparison between teacher and peer/self-reports: <0.001) and a larger group of victims (15.2%, p-value for comparison: <0.001). There were no statistically significant differences between teacher and child data in other bullying involvement groups.

**Table 1:** Child and maternal characteristics

	**Teacher report of bullying involvement (N = 3423)**		**Peer/self-report of bullying involvement (N = 1176)**	
**Child characteristics**	**N**	**% **^**a**^	**N**	**% **^**a**^
Mean age (years, SD in months)	3143	6.8 (3.03)	1176	7.6 (8.95)
Gender (% boys)	3422	50.6	1176	49.1
National origin	3400		1171	
Dutch	2146	63.1	767	65.5
Other Western	316	9.3	131	11.2
Non-western	938	27.6	273	23.3
Bullying involvement	3423		1176	
Uninvolved	2366	69.1	705	60.0
Bully	502	14.7	176	15.0
Victim	139	4.1	179	15.2
Bully-victim	416	12.1	116	9.8
Internalizing problems ^b^ (mean score, SD)	2892	5.07 (4.64)	997	4.79 (4.21)
Externalizing problems ^b^ (mean score, SD)	2908	10.60 (6.69)	1001	10.35 (6.55)
Day-care attendance (% not attending)	2872	33.4	992	29.4
TV exposure classes	3423		1176	
Low	603	17.6	243	20.7
Mid-low	1448	42.3	520	44.2
Mid-high	951	27.8	292	24.8
High	421	12.3	121	10.3
Exposure to violent TV/video content at age 5 years	2999		1053	
No	1434	47.8	499	47.4
Yes	1565	52.2	554	52.6
**Maternal characteristics**				
Mean age (years, SD)	3422	31.40 (4.75)	1176	32.09 (4.78)
Educational level	3241		1113	
Low	569	17.6	143	12.9
Mid-low	978	30.2	327	29.4
Mid-high	747	23.1	287	25.8
High	947	29.2	356	32.0
Monthly household income	2740		980	
<1200 (below social security level)	329	12.0	110	11.2
1200-2000 (average)	490	17.9	167	17.0
>2000 (modal)	1921	70.1	703	71.7
Marital status (% single)	3217	8.7	1114	9.1
Depression symptoms ^c^ (mean score, SD)	2972	0.13 (0.32)	1025	0.13 (0.31)
Parenting stress (mean score, SD) ^d^	2935	0.31 (0.30)	1007	0.32 (0.30)
Parity (% first-born)	3306	56.5	1134	56.2

^a^Unless otherwise indicated.

^b^Assessed with CBCL1½-5, the Dutch version of Child Behaviour Checklist.

^c^Measured with Brief Symptom Inventory.

^d^Parenting stress was measured with the Nijmeegse Ouderlijke Stress Index–Kort.

### Television viewing and bullying involvement

#### Latent classes

We identified latent classes of TV exposure at ages 2, 3, 4 and 5 years using LCA. The best fitting model, based on the smallest BIC, was a four-class model (see Additional file 1: Table S1). We considered BIC as a primary indicator of the model fit as this provides a reliable indication of the number of classes. Other model fit criteria were also acceptable, and although the LMR-LRT was still significant in the model with 5 classes, the statistical significance attenuated substantially (see Additional file 1: Table S1).

**Figure 2:**
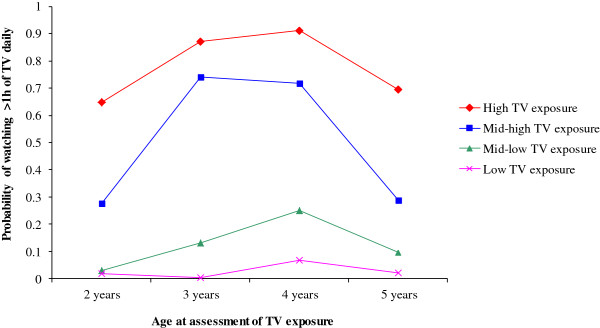
Latent classes of TV exposure conditional on probabilities of watching TV for >1 hour.

Latent classes, conditioned on children’s probabilities of watching TV for >1 hour daily at ages 2, 3, 4 and 5, are presented in Figure 2. Children with the highest probability of watching TV for >1 hour daily at all four ages, and children for whom this probability was the lowest were labeled as *'high’* and *'low’*, respectively. Two other classes of children were named *'mid-low’* and *'mid-high’*. In the mid-low, mid-high and high groups the probabilities of watching >1 hour of TV daily increased between ages 2-4 and were considerably lower at age 5 years. This decrease in the probabilities of TV viewing is probably due to the changes in daily routines and activities at age 4 years, as at this age children usually start preschool in the Netherlands. The distribution of children over the four TV exposure classes was very similar in teacher and child data (see Table 1). Children belonging to the latent class labeled as *'high exposure’* had also the highest probabilities of watching TV for >2 hours daily throughout ages 2-5, as it is shown in the Additional file 2: Figure S1.

#### TV exposure and bullying involvement

Association between TV exposure throughout ages 2-5 and bullying involvement (i.e. as a bully, victim, bully-victim vs. uninvolved) was examined using multinomial regression analyses (Table 2). First, we analyzed the association between TV latent classes and child bullying involvement using the teacher data. Univariate analyses (Table 2) showed that high TV exposure between ages 2-5 years was associated with a higher risk of being a bully (OR = 1.74, 95% CI: 1.22-2.50) or a victim (OR = 2.38, 95% CI: 1.33-4.28). Children in the mid-high and high TV exposure class were also more likely to be bully-victims. However, in the multivariate analyses, the associations between TV exposure classes and the risk of being a bully, victim or a bully-victim all attenuated and were no longer statistically significant. Next, we studied the association between television exposure classes and bullying involvement using the peer/self-reports (Table 2). In the univariate analyses, the mid-high and high television exposure classes were associated with an elevated risk of being a bully-victim (OR = 1.95, 95% CI: 0.99-3.83 and OR = 3.68, 95% CI: 1.75-7.74, respectively). Again, in the multivariate analyses, adjustment for child and maternal covariates substantially attenuated these effect estimates, and they were no longer statistically significant. No other associations between TV exposure and being a victim or a bully were found using the child-reported data.

**Table 2:** Latent classes of TV exposure between ages 2 and 5 years and bullying involvement in early elementary school

	**Teacher report (N = 3423)**				**Peer/self-report (N = 1176)**			
**TV exposure latent class**	**Unadjusted**		**Adjusted for covariates **^**a**^		**Unadjusted**		**Adjusted for covariates **^**a**^	
	**OR (95% CI)**	**p-value**	**OR (95% CI)**	**p-value**	**OR (95% CI)**	**p-value**	**OR (95% CI)**	**p-value**
	**Risk of being a bully **^b^							
Low	Ref		Ref		Ref		Ref	
Mid-low	1.07 (0.79-1.44)	0.66	1.00 (0.74-1.35)	0.99	0.85 (0.51-1.43)	0.54	0.68 (0.39-1.18)	0.17
Mid-high	1.35 (0.98-1.84)	0.07	1.15 (0.83-1.59)	0.42	1.28 (0.75-2.18)	0.37	0.86 (0.47-1.55)	0.61
High	1.74 (1.22-2.50)	0.002	1.27 (0.86-1.86)	0.23	1.33 (0.66-2.65)	0.43	0.71 (0.33-1.54)	0.39
	**Risk of being a victim **^b^							
Low	Ref		Ref		Ref		Ref	
Mid-low	1.17 (0.71-1.92)	0.54	1.16 (0.70-1.91)	0.57	0.91 (0.63-1.32)	0.64	0.88 (0.61-1.28)	0.51
Mid-high	1.11 (0.64-1.95)	0.71	1.02 (0.57-1.82)	0.96	0.98 (0.61-1.56)	0.93	0.83 (0.50-1.37)	0.47
High	2.38 (1.33-4.28)	0.004	1.80 (0.94-3.41)	0.07	1.10 (0.57-2.13)	0.77	0.85 (0.43-1.68)	0.63
	**Risk of being a bully-victim **^b^							
Low	Ref		Ref		Ref		Ref	
Mid-low	1.21 (0.88-1.65)	0.24	1.08 (0.79-1.48)	0.64	1.71 (0.88-3.32)	0.11	1.36 (0.70-2.65)	0.37
Mid-high	1.73 (1.25-2.40)	0.001	1.31 (0.93-1.85)	0.13	1.95 (0.99-3.83)	0.05	1.21 (0.59-2.46)	0.60
High	2.11 (1.42-3.13)	<0.001	1.35 (0.88-2.08)	0.17	3.68 (1.75-7.74)	0.001	1.60 (0.72-3.55)	0.25

Effect estimates are derived from multinomial regression analysis. Peer nomination scores were based on ratings by multiple peers.

^a^Adjusted for child gender, age, national origin, internalizing and externalizing problems and day-care attendance, and maternal age, parity, education, income, marital status, maternal symptoms of depression, parenting stress. ^b^Reference group: 'uninvolved in bullying’ children.

Additionally, we examined whether exposure to violent TV/video content at age 5 years was associated with children’s bullying involvement in the first grades of elementary school. The results of these analyses showed that exposure to violent content at age 5 years was associated with an increased risk of being a bully (OR = 1.27, 95% CI: 1.02-1.58) in early elementary school (see Additional file 3: Table S2).

We further explored which child or maternal factors explained the association between TV exposure classes and bullying involvement (Table 3). Using teacher reports, we examined the association between TV exposure class and bullying involvement, while separately adjusting the association for the following clusters of covariates: (a) *maternal socio-demographic factors*: maternal age, education, household income and marital status; and (b) *maternal psychosocial covariates*: depression symptoms and parenting stress; (c) *child socio-demographic characteristics*: gender, age, national origin, day-care attendance; (d) *child internalizing and externalizing problems*. We compared the unadjusted results to results obtained after adjustment for each of the separate groups of covariates (Table 3). Using teacher data, we found that the association between TV exposure class and the risk of being a *bully* or a *bully-victim* was largely confounded by maternal socio-demographic characteristics (for high exposure class OR_bully_ = 1.30, 95% CI: 0.90-1.90 and OR_bully-victim_ = 1.39, 95% CI: 0.91-2.11). Additional analyses with individual covariates of this group of covariates showed that the association attenuated mainly due to *maternal age, educational level and household income*. Similar analyses in child-reported data also showed that the effect of television viewing that was found for bully-victims was confounded by these maternal socio-demographic characteristics (see Additional file 4: Table S3). Adjustment of the association for the other clusters of covariates also resulted in an attenuation of the univariate effect estimates, however that attenuation of the effects was smaller than that after controlling for the maternal socio-demographic covariates. Adjusting the analyses jointly for all covariates resulted in the strongest attenuation of the effects, as can be seen by comparing the separate adjustment models in Table 3 with the fully adjusted model presented in Table 2.

**Table 3:** **Confounding patterns of the association between TV exposure between ages 2 and 5 years and *teacher report *** **of bullying involvement in early elementary school**

	**Teacher report (N = 3423)**									
**TV exposure latent class**	**Model 1: Unadjusted**		**Model 1 adjusted for maternal socio-demographic covariates **^**a**^		**Model 1 adjusted for maternal psychosocial covariates **^**b**^		**Model 1 adjusted for child socio-demographic covariates **^**c**^		**Model 1 adjusted for child internalizing and externalizing problems **^**d**^	
		**OR (95% CI)**	**p-value**	**OR (95% CI)**	**p-value**	**OR (95% CI)**	**p-value**	**OR (95% CI)**	**p-value**	**OR (95% CI)**	**p-value**
	**Risk of being a bully**									
Low	Ref		Ref		Ref		Ref		Ref	
Mid-low	1.07 (0.79-1.44)	0.66	1.00 (0.74-1.35)	1.00	1.07 (0.80-1.44)	0.65	1.05 (0.78-1.42)	0.74	1.05 (0.78-1.42)	0.73
Mid-high	1.35 (0.98-1.84)	0.07	1.16 (0.84-1.60)	0.37	1.33 (0.97-1.82)	0.08	1.27 (0.92-1.76)	0.14	1.32 (0.96-1.81)	0.09
High	1.74 (1.22-2.50)	0.002	1.30 (0.90-1.90)	0.16	1.70 (1.18-2.44)	0.004	1.50 (1.03-2.21)	0.04	1.71 (1.19-2.48)	0.004
	**Risk of being a victim**									
Low	Ref		Ref		Ref		Ref		Ref	
Mid-low	1.17 (0.71-1.92)	0.54	1.13 (0.68-1.86)	0.63	1.16 (0.71-1.91)	0.55	1.13 (0.69-1.87)	0.62	1.17 (0.71-1.92)	0.54
Mid-high	1.11 (0.64-1.95)	0.71	1.03 (0.58-1.84)	0.91	1.09 (0.62-1.92)	0.77	1.00 (0.56-1.76)	0.99	1.09 (0.61-1.92)	0.77
High	2.38 (1.33-4.28)	0.004	1.93 (1.03-3.66)	0.04	2.27 (1.25-4.12)	0.007	1.94 (1.02-3.44)	0.04	2.20 (1.21-3.99)	0.01
	**Risk of being a bully-victim**									
Low	Ref		Ref		Ref		Ref		Ref	
Mid-low	1.21 (0.88-1.65)	0.24	1.08 (0.79-1.48)	0.63	1.22 (0.89-1.66)	0.21	1.17 (0.86-1.60)	0.32	1.18 (0.87-1.62)	0.28
Mid-high	1.73 (1.25-2.40)	0.001	1.33 (0.94-1.87)	0.11	1.68 (1.21-2.33)	0.002	1.61 (1.16-2.24)	0.005	1.68 (1.21-2.33)	0.002
High	2.11 (1.42-3.13)	<0.001	1.39 (0.91-2.11)	0.13	1.98 (1.33-2.95)	0.001	1.79 (1.19-2.71)	0.005	2.01 (1.35-3.00)	0.001

Effect estimates are derived from multinomial regression analysis. Reference group: 'uninvolved in bullying’ children.

^a^Adjusted for maternal age, education, income and marital status. ^b^Adjusted for parity, maternal symptoms of depression and parenting stress. ^c^Adjusted for child gender, age, national origin, day-care attendance. ^d^Adjusted for child internalizing and externalizing problems. For fully adjusted model see Table 2.

Finally, we additionally examined the association between TV viewing and bullying involvement by analyzing the separate exposure measurements of television viewing at each of the four different ages. As shown in Additional file 5: Tables S4, Additional file 6: Table S5, Additional file 7: Table S6 and Additional file 8: Table S7, we found no effect of television viewing on bullying or victimization at any of the ages.

## Discussion

We studied child television exposure throughout ages 2-5 years in relation to teacher- and peer/self-reports of bullying involvement in early elementary school. In the univariate analyses, we observed an association between high television exposure and the risk of being involved in school bullying; however, this association attenuated after adjustment for the covariates. This finding was consistent in both teacher- and peer/self-reported data. These results differ from the findings of two other prospective studies: Pagani et al [25], who found that each extra hour of television viewing at age 2.4 years led to 10% unit increase in peer victimization at age 10 years; and Zimmerman et al [24], who reported that each additional hour of television viewed per day at age 4 years was significantly associated with an odds ratio of 1.06 for bullying at age 6-11 years. The effect estimates reported in those studies were relatively small, yet statistically significant and, in contrast to our findings, remained significant after adjustment for child and family factors.

Several possible explanations of the discrepancies between our findings and the results of earlier studies should be considered. Our measure of exposure was different. Also, we showed that there are specific covariates (e.g. maternal age, educational level and family income) that strongly confound the association between television viewing and bullying involvement. Similarly, other studies showed that these family characteristics are related to both – bullying [2] and child television viewing [39]. Unlike in the studies of Pagani and Zimmerman [24,25], we adjusted our analyses for child internalizing and externalizing problems at age 18 months. Child behavioral problems may be important potential confounding factors because television viewing is known to be associated with child externalizing problems [22]; and child internalizing and externalizing problems are associated with bullying involvement [7]. Furthermore, the adjustment for early age behavioral problems helped us eliminate a concern that children may watch TV as a result of their pre-existing problems, as parents of children with behavioral problems may be more inclined to allow TV viewing [40,41]. Our findings show that children’s internalizing and externalizing problems do, to some extent, confound the association between TV exposure and bullying involvement, as the effect estimates decreased after adjustment for child problem behavior (as shown in Table 3). However, the most substantial decrease in effect estimates resulted from the adjustment for maternal socio-demographic variables (Table 3), demonstrating that both children’s high television exposure and bullying involvement are strongly related to such underlying factors as maternal age, educational level and income.

In our study, children’s exposure to violent TV/video content at age 5 years was associated with the risk of being a bully, but not with the risk of being a victim or a bully-victim. Several possible explanations of this finding should be considered. The content-based theories suggest that children learn from observing violence, which is thought to effect children’s aggressive behavior [23,42]. Following this approach, exposure to violent content may trigger the aggressive behavior of bullies. Possibly, observing this effect in the group of bullies, but not in the group of bully-victims could be due to different effects of violent content on proactive vs reactive aggression. We may speculate that the exposure to violence has a stronger effect on proactive aggression of bullies rather than on reactive aggression of bully-victims. Yet, this interpretation needs further in depth, possibly qualitative examination. Finally, due to the cross-sectional nature of this specific analysis, we cannot infer causality or establish the direction of the association (i.e. the data were collected prospectively, however the age difference between the assessments was not large and children’s bullying involvement was measured only once, precluding adjustment for bullying involvement at baseline). While it is plausible that viewing of violent content leads to bullying behavior, it is also possible that aggressive children, who are involved in bullying at school-entry age have a stronger preference for viewing violent TV/video programs [17,43].

In order to avoid the problem of shared method variance and possible reporter bias, multiple informants were used in our study. Relying on teacher or parent as the only informant may be insufficient, and complementary information can be obtained from peers who, compared to a teacher or a parent, are often more aware of peer relations in a class. The peer/self-report of bullying involvement used in our study is a composite measure of the self-reported victimization and the peers’ reports of bullying. Allowing all the children in a class rate one another with regard to bullying involvement provides a reliable measure of bullying involvement from the perspective of the entire group. Such approach eliminates possible bias that can be introduced by the use of only teacher or parent report. In the previous studies, bullying assessment was confined to maternal [24] or teacher [25] report only. Our findings show that the effects of television viewing on child-reported bullying were, if anything, smaller than the effects found in teacher data; although, for the group of bully-victims the strength of the effect estimates was very similar in the teacher and child data.

In sum, our findings provide some support for the content-based theory, as watching violent television content at age 5 years was associated with the teacher report of bullying involvement at age 7 years. However, this finding should be replicated using longitudinal data in order to determine the direction of the association. Our findings further suggest that the observed negative effects of the television exposure time on bullying involvement, reasoned to occur due to excessive TV viewing according the displacement theory, are confounded by maternal and child socio-demographic characteristics. Maternal socio-demographic characteristics (i.e. maternal age, education, income, marital status) appear to be the underlying factors associated with both children’s excessive television exposure and bullying involvement.

The relation between these maternal socio-demographic characteristics and child behavior – i.e. an excessive television viewing, bullying involvement – has been reported in earlier studies [2,39,44]. A young age, low socioeconomic background and being a single parent are associated with negative outcomes in child development. Children of younger mothers are more likely to show developmental problems, e.g. behavioral problems, which is likely due to these children being brought up in a rather disadvantaged environment [45]. Fergusson and Lynskey [45] explain that children born to younger mothers are brought up in families that are socially and educationally disadvantaged, and also less nurturing and more unstable. Family’s socioeconomic disadvantage is associated with children’s emotional and behavioral problems, either directly (e.g. stress-induced) or through parenting practices [46,47]. Socio-demographic characteristics, like parental educational level and income, also reflect various resources and skills, including intellect, literacy, problem-solving skills, and norms and values of a parent [48,49], that can influence children’s social development and behavior through parental rearing practices [50]. Similarly, being a single parent may negatively affect the upbringing practices and parent-child interactions through its inherent stress and reduced parental control over child’s behavior [2]. Importantly, having understood the role of these socio-demographic characteristics, they can be used as indicators in identifying the vulnerable groups of children at risk of behavioral problems. These vulnerable groups can then be targeted by prevention and intervention programs aimed at prevention of excessive media use and bullying involvement. For instance, future studies could examine whether intervention programs aiming to enhance knowledge, problem-solving skills and parenting practices of socioeconomically disadvantaged parents could yield positive effects with regard to both outcomes – media exposure and peer interactions of young children.

Our study’s major strengths are the use of multiple reporters and repeated assessments of the exposure at preschool age. Yet, several limitations of the present study should be discussed. First, we used parental report of television exposure which is inferior to observational or diary-based measures. However, in large data collections required for population-based studies such as the present one, (parental) questionnaires are the most feasible assessment method of child television exposure. Second, our measure of children’s exposure to violent content was not very detailed. As already discussed above, this measure was assessed only once, when children were 5 years old, and thus it did not allow longitudinal examination of the relation. Importantly, our measure of content contained information on whether or not the children watched violent content on TV/video, but not on the duration or the actual content of the programs watched. The exposure to specific television programs may be associated differently with bullying involvement than the duration of such TV exposure as a whole. Thus, an objective and more detailed measure of the violent content could have resulted in a stronger association with children’s bullying behaviors. Future studies should also consider the role of other important factors, such as children's exposure to aggression or abuse in real life. Also, using continuous measure of TV viewing can offer more precision. We used categorical measures of exposure; however, these measures had multiple categories that reflected daily hours of TV viewing, which in combination with multiple assessments over time were likely to provide sufficient information on children’s TV viewing. Finally, we did not have information on children’s bullying involvement prior to school entry, thus we were not able to examine whether television viewing could predict incidence of bullying involvement.

## Conclusions

In summary, our findings demonstrate that a child’s risk of bullying involvement in early elementary school that is associated with preschool television exposure is largely explained by confounding factors – primarily maternal socio-demographic characteristics. Our results suggest that social disadvantage, as indicated by the socioeconomic factors such as low income and lower educational level, may pose the actual risk for high television viewing at preschool age and for bullying involvement in early elementary school. This should be further examined in future studies.

## Abbreviations

TV: Television; CBCL1½ - 5: Child behavior checklist for toddlers; OR: Odds ratio; CI: Confidence interval; LCA: Latent class analyses; BIC: Bayesian information criterion; LMR-LRT: Lo-mendel-rubin likelihood ratio-test.

## Competing interests

Dr. Frank C. Verhulst is a contributing editor of *the Achenbach System of Empirically Based Assessment*, from which he receives remuneration. For the other authors, no potentially competing financial interest is declared.

## Authors’ contributions

MV conceptualized the study, performed statistical analyses and drafted the manuscript. HT, RV, VWVJ, HR, AH, CM and WJ made substantial contributions to the acquisition and interpretation of the data. PWJ contributed to the design of the study and supervised the statistical analyses and writing of the manuscript. HT, RV, and FCV made substantial contributions to the conception and design of the study. HT, HR, VWVJ, CM, WJ and PWJ were also involved in the interpretation of the data. HT made a substantial contribution to the conception and design of the study, interpretation of the data and supervised the drafting of the manuscript. All authors critically revised the manuscript and approved the final version of the manuscript.

## Pre-publication history

The pre-publication history for this paper can be accessed here:

http://www.biomedcentral.com/1471-2458/14/157/prepub

## Supplementary Material

Additional file 1: Table S1 LCA models characteristics (N = 5389). Click here for file

Additional file 2: Figure S1 Latent classes of TV exposure conditional on probabilities of watching TV >2 h. Click here for file

Additional file 3: Table S2 Exposure to violent TV/video content and bullying involvement in early elementary school. Click here for file

Additional file 4: Table S3 Confounding patterns of the association between TV exposure at 2-5 years and *peer/self-reported* bullying involvement in early elementary school. Click here for file

Additional file 5: Table S4 TV exposure *at age 2 years* and bullying involvement in early elementary school. Click here for file

Additional file 6: Table S5 TV exposure *at age 3 years* and bullying involvement in early elementary school. Click here for file

Additional file 7: Table S6 TV exposure *at age 4 years* and bullying involvement at early elementary school. Click here for file

Additional file 8: Table S7 TV exposure *at age 5 years* and bullying involvement at early elementary school. Click here for file
