# Supplementary material for: Television viewing through ages 2-5 years and bullying involvement in early elementary school
Source: BMC Public Health. 2014 Feb 12;14:157. doi: 10.1186/1471-2458-14-157 (PMC3944918; doi:10.1186/1471-2458-14-157)
Supplement: Additional file 1: Table S1 — LCA models characteristics (N = 5389). [file 1471-2458-14-157-S1.doc]

**Table S1**

**LCA models characteristics (N=5389).**

| **Number of classes** | **BIC****^a^** | **p-value for LMR-LRT**^b^ |
| --- | --- | --- |
| 1 | 43467.1 | NA |
| 2 | 40513.4 | <0.0001 |
| 3 | 39792.6 | <0.0001 |
| **4** | **39736.0** | <0.0001 |
| 5 | 39814.8 | 0.0096 |

^a^A lower value of BIC indicates a better fit of the model.

^b^The LMR-LRT compares the fit of two models that differ by one class. A non-significant value indicates that the model with one class less is preferred.
