# Supplementary material for: Television viewing through ages 2-5 years and bullying involvement in early elementary school
Source: BMC Public Health. 2014 Feb 12;14:157. doi: 10.1186/1471-2458-14-157 (PMC3944918; doi:10.1186/1471-2458-14-157)
Supplement: Additional file 2: Figure S1 — Latent classes of TV exposure conditional on probabilities of watching TV >2 h. [file 1471-2458-14-157-S2.doc]

**Figure S1**

**Latent classes of TV exposure conditional on probabilities of watching TV >2h**
