# Supplementary material for: Television viewing through ages 2-5 years and bullying involvement in early elementary school
Source: BMC Public Health. 2014 Feb 12;14:157. doi: 10.1186/1471-2458-14-157 (PMC3944918; doi:10.1186/1471-2458-14-157)
Supplement: Additional file 3: Table S2 — Exposure to violent TV/video content and bullying involvement in early elementary school. [file 1471-2458-14-157-S3.doc]

**Table S2**

**Exposure to violent TV/video content and bullying involvement in early elementary school**

|  | **Teacher report (N=2999)** | | | | | **Peer/self-report (N=1053)** | | | | |
| --- | --- | --- | --- | --- | --- | --- | --- | --- | --- | --- |
| **Exposure to violent TV/video content at age 5 years** | Unadjusted | | Adjusted for covariates ^a^ | |  | | Unadjusted | | Adjusted for covariates ^a^ | |
|  | OR (95% CI) | p-value | OR (95% CI) | p-value |  | | OR (95% CI) | p-value | OR (95% CI) | p-value |
|  |  |  |  |  |  |  |  |  |  |  |
|  | **Risk of being a bully** ^b^ | | | | | | | | | |
| No | Ref |  | Ref |  |  | | Ref |  | Ref |  |
| Yes | **1.28** (1.04-1.59) | 0.02 | **1.27** (1.02-1.58)^c^ | 0.03 |  | | 1.30 (0.91-1.86) | 0.15 | 1.10 (0.74-1.63) | 0.65 |
|  |  |  |  |  |  | |  |  |  |  |
|  | **Risk of being a victim** ^b^ | | | | | | | | | |
| No | Ref |  | Ref |  |  | | Ref |  | Ref |  |
| Yes | 0.93 (0.64-1.35) | 0.69 | 0.93 (0.63-1.38) | 0.71 |  | | 1.10 (0.77-1.59) | 0.59 | 1.26 (0.85-1.89) | 0.25 |
|  |  |  |  |  |  | |  |  |  |  |
|  | **Risk of being a bully-victim** ^b^ | | | | | | | | | |
| No | Ref |  | Ref |  |  | | Ref |  | Ref |  |
| Yes | 1.12 (0.89-1.42) | 0.34 | 1.07 (0.84-1.38) | 0.58 |  | | 1.16 (0.74-1.82) | 0.67 | 1.02 (0.63-1.64) | 0.95 |

Effect estimates are derived from the multinomial regression analysis. Peer nomination scores were based on ratings by multiple peers.

^a^ Adjusted for child gender, age, national origin, internalizing and externalizing problems and day-care attendance, and maternal age, parity, education, income, marital status, maternal symptoms of depression, parenting stress. ^b^ Reference group: ‘uninvolved in bullying’ children.

^c^ If additionally adjusted for the TV exposure classes: OR=1.26 (95%CI: 1.01-1.57), p=0.04.
