# Supplementary material for: Television viewing through ages 2-5 years and bullying involvement in early elementary school
Source: BMC Public Health. 2014 Feb 12;14:157. doi: 10.1186/1471-2458-14-157 (PMC3944918; doi:10.1186/1471-2458-14-157)
Supplement: Additional file 4: Table S3 — Confounding patterns of the association between TV exposure at 2-5 years and peer/self-reported bullying involvement in early elementary school. [file 1471-2458-14-157-S4.doc]

# Table S3

**Confounding patterns of the association between TV exposure at 2-5 years and *peer/self-reported* bullying involvement in early elementary school**

|  | **Peer/self-report (N=1176)** | | | | | | | | | | |
| --- | --- | --- | --- | --- | --- | --- | --- | --- | --- | --- | --- |
| **TV exposure latent class** | Model 1: Unadjusted | | Model 1 adjusted for maternal socio-demographic covariates ^a^ | | Model 1 adjusted for maternal psychosocial covariates ^b^ | | Model 1 adjusted for child socio-demographic covariates ^c^ | | Model 1 adjusted for child internalizing and externalizing problems ^d^ | | |
|  | OR (95% CI) | p-value | OR (95% CI) | p-value | OR (95% CI) | p-value | OR (95% CI) | p-value | OR (95% CI) | p-value | |
|  |  |  |  |  |  |  |  |  |  |  |  |
|  | **Risk of being a bully** | | | | | | | | | | |
| Low | Ref |  | Ref |  | Ref |  | Ref |  | Ref | |  |
| Mid-low | 0.85 (0.51-1.43) | 0.54 | 0.72 (0.42-1.21) | 0.21 | 0.82 (0.49-1.39) | 0.46 | 0.77 (0.45-1.33) | 0.35 | 0.84 (0.50-1.42) | | 0.52 |
| Mid-high | 1.28 (0.75-2.18) | 0.37 | 0.90 (0.52-1.58) | 0.72 | 1.20 (0.70-2.08) | 0.51 | 1.07 (0.60-1.91) | 0.82 | 1.24 (0.73-2.13) | | 0.43 |
| High | 1.33 (0.66-2.65) | 0.43 | 0.79 (0.38-1.65) | 0.53 | 1.19 (0.59-2.40) | 0.63 | 0.95 (0.45-2.01) | 0.89 | 1.25 (0.62-2.50) | | 0.53 |
|  |  |  |  |  |  |  |  |  |  | | |
|  | **Risk of being a victim** | | | | | | | | | | |
| Low | Ref |  | Ref |  | Ref |  | Ref |  | Ref | |  |
| Mid-low | 0.91 (0.63-1.32) | 0.64 | 0.87 (0.60-1.26) | 0.45 | 0.90 (0.62-1.30) | 0.57 | 0.92 (0.63-1.34) | 0.68 | 0.91 (0.63-1.32) | | 0.63 |
| Mid-high | 0.98 (0.61-1.56) | 0.93 | 0.81 (0.51-1.30) | 0.39 | 0.98 (0.62-1.56) | 0.93 | 0.96 (0.59-1.57) | 0.87 | 0.98 (0.61-1.56) | | 0.85 |
| High | 1.10 (0.57-2.13) | 0.77 | 0.81 (0.42-1.57) | 0.53 | 1.10 (0.57-2.12) | 0.77 | 1.12 (0.58-2.17) | 0.74 | 1.10 (0.57-2.13) | | 0.77 |
|  |  |  |  |  |  |  |  |  |  | | |
|  | **Risk of being a bully-victim** | | | | | | | | | | |
| Low | Ref |  | Ref |  | Ref |  | Ref |  | Ref | |  |
| Mid-low | 1.71 (0.88-3.32) | 0.11 | 1.39 (0.72-2.68) | 0.33 | 1.65 (0.84-3.23) | 0.14 | 1.54 (0.78-3.03) | 0.21 | 1.70 (0.87-3.31) | | 0.12 |
| Mid-high | **1.95** (0.99-3.83) | 0.05 | 1.18 (0.60-2.34) | 0.64 | 1.87 (0.95-3.69) | 0.07 | 1.57 (0.78-3.17) | 0.20 | 1.91 (0.97-3.74) | | 0.06 |
| High | **3.68** (1.75-7.74) | 0.001 | 1.64 (0.73-3.64) | 0.23 | **3.47** (1.65-7.32) | 0.001 | **2.39** (1.09-5.26) | 0.03 | **3.51** (1.67-7.41) | | 0.001 |

Reference group: ‘uninvolved in bullying’ children. Peer nomination scores were based on ratings by multiple peers.

^a^ Adjusted for maternal age, education, income and marital status. ^b^ Adjusted for parity, maternal symptoms of depression and parenting stress. ^c^ Adjusted for child gender, age, national origin, day-care attendance. ^d^ Adjusted for child internalizing and externalizing problems. For fully adjusted model see Table 2.
