# Supplementary material for: Television viewing through ages 2-5 years and bullying involvement in early elementary school
Source: BMC Public Health. 2014 Feb 12;14:157. doi: 10.1186/1471-2458-14-157 (PMC3944918; doi:10.1186/1471-2458-14-157)
Supplement: Additional file 5: Table S4 — TV exposure at age 2 years and bullying involvement in early elementary school. [file 1471-2458-14-157-S5.doc]

**Table S4**

**TV exposure *at age 2 years* and bullying involvement in early elementary school**

|  | **Teacher report (N=3111)** | | | **Peer/self-report (N=1067)** | | | | | | |
| --- | --- | --- | --- | --- | --- | --- | --- | --- | --- | --- |
| **TV exposure at age 2 years** | Adjusted for covariates ^a^ | | |  | Adjusted for covariates ^a^ | | | | | |
|  |  | OR (95% CI) | p-value |  |  | | | OR (95% CI) | p-value | |
|  |  |  |  |  |  |  |  |  |  |  |
|  | **Risk of being a bully** | | | | | | | | | |
| Never |  | Ref |  |  | | | Ref | | |  |
| <0.5 hour |  | 1.29 (0.80-2.09) | 0.29 |  |  |  | 1.18 (0.54-2.58) | | | 0.63 |
| 0.5-1 hour |  | 1.12 (0.69-1.82) | 0.65 |  |  |  | 0.98 (0.42-2.27) | | | 0.20 |
| >1 hour |  | 1.34 (0.80-2.27) | 0.27 |  |  |  | 1.22 (0.48-3.09) | | | 0.84 |
|  |  |  |  |  |  |  |  |  |  |  |
|  | **Risk of being a victim** | | | | | | | | | |
| Never |  | Ref |  |  | | | Ref | | |  |
| <0.5 hour |  | 2.69 (0.81-8.97) | 0.11 |  |  |  | 1.06 (0.54-2.07) | | | 0.86 |
| 0.5-1 hour |  | 1.97 (0.58-6.64) | 0.28 |  |  |  | 0.92 (0.46-1.86) | | | 0.82 |
| >1 hour |  | **3.38** (0.99-11.56) | 0.05 |  |  |  | 0.89 (0.39-2.06) | | | 0.79 |
|  |  |  |  |  |  |  |  |  |  |  |
|  | **Risk of being a bully-victim** | | | | | | | | | |
| Never |  | Ref |  |  | | Ref | | | |  |
| <0.5 hour |  | 0.64 (0.39-1.04) | 0.07 |  | | 1.72 (0.52-5.75) | | | | 0.38 |
| 0.5-1 hour |  | 0.82 (0.50-1.34) | 0.43 |  | | 1.82 (0.55-6.02) | | | | 0.33 |
| >1 hour |  | 0.85 (0.50-1.42) | 0.53 |  | | 1.40 (0.39-5.02) | | | | 0.60 |

Reference group: ‘uninvolved in bullying’ children. Peer nomination scores were based on ratings by multiple peers.

^a^ Adjusted for child gender, age, national origin, internalizing and externalizing problems and day-care attendance, and maternal age, parity, education, income, marital status, maternal symptoms of depression, parenting stress.
