# Supplementary material for: Television viewing through ages 2-5 years and bullying involvement in early elementary school
Source: BMC Public Health. 2014 Feb 12;14:157. doi: 10.1186/1471-2458-14-157 (PMC3944918; doi:10.1186/1471-2458-14-157)
Supplement: Additional file 6: Table S5 — TV exposure at age 3 years and bullying involvement in early elementary school. [file 1471-2458-14-157-S6.doc]

# Table S5

**TV exposure *at age 3 years* and bullying involvement in early elementary school**

|  | **Teacher report (N=2938)** | | | **Peer/self-report (N=1016)** | | | | | | |
| --- | --- | --- | --- | --- | --- | --- | --- | --- | --- | --- |
| **TV exposure at age 3 years** | Adjusted for covariates ^a^ | | |  | Adjusted for covariates ^a^ | | | | | |
|  |  | OR (95% CI) | p-value |  |  | | | OR (95% CI) | p-value | |
|  |  |  |  |  |  |  |  |  |  |  |
|  | **Risk of being a bully** | | | | | | | | | |
| Never and <0.5 hour |  | Ref |  |  | | | Ref | | |  |
| <0.5-1 hour |  | 0.95 (0.71-1.26) | 0.71 |  |  |  | 0.66 (0.39-1.12) | | | 0.13 |
| 1-2 hours |  | 1.09 (0.80-1.50) | 0.57 |  |  |  | 0.85 (0.48-1.51) | | | 0.57 |
| >2 hours |  | 1.00 (0.65-1.53) | 0.98 |  |  |  | 0.72 (0.32-1.59) | | | 0.42 |
|  |  |  |  |  |  |  |  |  |  |  |
|  | **Risk of being a victim** | | | | | | | | | |
| Never and <0.5 hour |  | Ref |  |  | | | Ref | | |  |
| <0.5-1 hour |  | 1.51 (0.89-2.56) | 0.12 |  |  |  | 0.80 (0.53-1.21) | | | 0.29 |
| 1-2 hours |  | 1.36 (0.75-2.45) | 0.31 |  |  |  | 0.87 (0.51-1.48) | | | 0.61 |
| >2 hours |  | 1.80 (0.86-3.75) | 0.12 |  |  |  | 0.61 (0.24-1.55) | | | 0.30 |
|  |  |  |  |  |  |  |  |  |  |  |
|  | **Risk of being a bully-victim** | | | | | | | | | |
| Never and <0.5 hour |  | Ref |  |  | | Ref | | | |  |
| <0.5-1 hour |  | 0.97 (0.71-1.31) | 0.83 |  |  | 1.40 (0.77-2.55) | | | | 0.27 |
| 1-2 hours |  | 1.30 (0.93-1.82) | 0.12 |  |  | 1.32 (0.65-2.69) | | | | 0.45 |
| >2 hours |  | 1.17 (0.74-1.87) | 0.50 |  |  | 2.06 (0.81-5.25) | | | | 0.13 |

Reference group: ‘uninvolved in bullying’ children. Peer nomination scores were based on ratings by multiple peers.

^a^ Adjusted for child gender, age, national origin, internalizing and externalizing problems and day-care attendance, and maternal age, parity, education, income, marital status, maternal symptoms of depression, parenting stress.
