# Supplementary material for: Television viewing through ages 2-5 years and bullying involvement in early elementary school
Source: BMC Public Health. 2014 Feb 12;14:157. doi: 10.1186/1471-2458-14-157 (PMC3944918; doi:10.1186/1471-2458-14-157)
Supplement: Additional file 7: Table S6 — TV exposure at age 4 years and bullying involvement at early elementary school. [file 1471-2458-14-157-S7.doc]

# Table S6

**TV exposure *at age 4 years* and bullying involvement at early elementary school**

|  | **Teacher report (N=2967)** | | | **Peer/self-report (N=1036)** | | | | | | |
| --- | --- | --- | --- | --- | --- | --- | --- | --- | --- | --- |
| **TV exposure at age 4 years** | Adjusted for covariates ^a^ | | |  | Adjusted for covariates ^a^ | | | | | |
|  |  | OR (95% CI) | p-value |  |  | | | OR (95% CI) | p-value | |
|  |  |  |  |  |  |  |  |  |  |  |
|  | **Risk of being a bully** | | | | | | | | | |
| <0.5 hour |  | Ref |  |  | | | Ref | | |  |
| 0.5-1 hour |  | 0.80 (0.54-1.18) | 0.27 |  |  |  | 0.84 (0.45-1.56) | | | 0.58 |
| 1-2 hours |  | 0.80 (0.53-1.21) | 0.30 |  |  |  | 1.15 (0.58-2.31) | | | 0.69 |
| >2 hours |  | 0.99 (0.61-1.63) | 0.98 |  |  |  | 0.97 (0.41-2.29) | | | 0.95 |
|  |  |  |  |  |  |  |  |  |  |  |
|  | **Risk of being a victim** | | | | | | | | | |
| <0.5 hour |  | Ref |  |  | | | Ref | | |  |
| 0.5-1 hour |  | 0.70 (0.40-1.22) | 0.23 |  |  |  | **2.03** (1.02 -4.07) | | | 0.04 |
| 1-2 hours |  | 0.63 (0.33-1.20) | 0.56 |  |  |  | 1.46 (0.72-2.98) | | | 0.29 |
| >2 hours |  | 1.34 (0.67-2.69) | 0.40 |  |  |  | 2.12 (0.86-5.22) | | | 0.10 |
|  |  |  |  |  |  |  |  |  |  |  |
|  | **Risk of being a bully-victim** | | | | | | | | | |
| <0.5 hour |  | Ref |  |  | | Ref | | | |  |
| 0.5-1 hour |  | 1.03 (0.66-1.60) | 0.90 |  |  | 0.59 (0.33-1.06) | | | | 0.08 |
| 1-2 hours |  | 1.10 (0.69-1.75) | 0.68 |  |  | 0.61 (0.32-1.16) | | | | 0.13 |
| >2 hours |  | 1.28 (0.73-2.24) | 0.39 |  |  | 0.85 (0.38-1.93) | | | | 0.70 |

Reference group: ‘uninvolved in bullying’ children. Peer nomination scores were based on ratings by multiple peers.

^a^ Adjusted for child gender, age, national origin, internalizing and externalizing problems and day-care attendance, and maternal age, parity, education, income, marital status, maternal symptoms of depression, parenting stress.
