# Supplementary material for: Television viewing through ages 2-5 years and bullying involvement in early elementary school
Source: BMC Public Health. 2014 Feb 12;14:157. doi: 10.1186/1471-2458-14-157 (PMC3944918; doi:10.1186/1471-2458-14-157)
Supplement: Additional file 8: Table S7 — TV exposure at age 5 years and bullying involvement at early elementary school. [file 1471-2458-14-157-S8.doc]

# Table S7

**TV exposure *at age 5 years* and bullying involvement at early elementary school**

|  | **Teacher report (N=3124)** | | | **Peer/self-report (N=1091)** | | | | | | |
| --- | --- | --- | --- | --- | --- | --- | --- | --- | --- | --- |
| **TV exposure at age 5 years** | Adjusted for covariates ^a^ | | |  | Adjusted for covariates ^a^ | | | | | |
|  |  | OR (95% CI) | p-value |  |  | | | OR (95% CI) | p-value | |
|  |  |  |  |  |  |  |  |  |  |  |
|  | **Risk of being a bully** | | | | | | | | | |
| <0.5 hour |  | Ref |  |  | | Ref | | | |  |
| 0.5-1 hour |  | 0.98 (0.63-1.51) | 0.91 |  |  | 0.56 (0.29-1.10) | | | | 0.33 |
| 1-2 hours |  | 0.90 (0.55-1.45) | 0.66 |  |  | 0.64 (0.29-1.42) | | | | 0.10 |
| >2 hours |  | 1.07 (0.52-2.21) | 0.85 |  |  | 1.40 (0.35-5.65) | | | | 0.66 |
|  |  |  |  |  |  |  |  |  |  |  |
|  | **Risk of being a victim** | | | | | | | | | |
| <0.5 hour |  | Ref |  |  | | Ref | | | |  |
| 0.5-1 hour |  | 0.65 (0.32-1.30) | 0.22 |  |  | 1.00 (0.49-2.08) | | | | 0.99 |
| 1-2 hours |  | 0.88 (0.41-1.88) | 0.75 |  |  | 1.28 (0.55-2.99) | | | | 0.57 |
| >2 hours |  | 0.75 (0.22-2.50) | 0.63 |  |  | 1.34 (0.22-8.05) | | | | 0.75 |
|  |  |  |  |  |  |  |  |  |  |  |
|  | **Risk of being a bully-victim** | | | | | | | | | |
| <0.5 hour |  | Ref |  |  | | | Ref | | |  |
| 0.5-1 hour |  | 1.59 (0.88-2.85) | 0.12 |  | | | 0.73 (0.30-1.78) | | | 0.50 |
| 1-2 hours |  | 1.68 (0.91-3.10) | 0.10 |  | | | 1.07 (0.38-3.00) | | | 0.90 |
| >2 hours |  | 1.21 (0.48-3.04) | 0.69 |  | | | 1.94 (0.34-10.94) | | | 0.46 |

Reference group: ‘uninvolved in bullying’ children. Peer nomination scores were based on ratings by multiple peers.

^a^ Adjusted for child gender, age, national origin, internalizing and externalizing problems and day-care attendance, and maternal age, parity, education, income, marital status, maternal symptoms of depression, parenting stress.
